# Loss of responsiveness on reinstatement of antidepressants after treatment interruption – A systematic review

**DOI:** 10.1177/02698811251364388

**Published:** 2025-09-13

**Authors:** Ninoslav Majkic, David Taylor

**Affiliations:** 1 Pharmacy Department, South London and Maudsley NHS Foundation Trust, London, UK; 2 Institute of Pharmaceutical Science, King’s College London, London, UK

**Keywords:** Antidepressants, systematic review, intermittent adherence, stopping

## Abstract

**Background:**

The societal burden of depression continues to increase despite the greater use of antidepressants. It is not clear why wider antidepressant prescribing has not reduced the impact of depression at a population level. One possible explanation is that intermittent use of antidepressants at an individual level might reduce responsiveness to antidepressants.

**Methods:**

We searched EMBASE and PubMed from the beginning of records to June 2024 for articles describing loss of response to antidepressants (in any psychiatric condition) occurring as a result of interruption in treatment. We did not restrict our search with respect to language or date.

**Results:**

We found 6869 articles of potential interest, of which 5360 were excluded after initial screening by title, and 1453 were excluded as duplicates. We ultimately included 12 studies that provided data on 594 participants. Non-response was reported in 4%–57% of people who stopped and restarted antidepressant treatment that was previously effective.

**Conclusion:**

Non-continuous consumption of antidepressants leads to a loss of responsiveness in an important proportion of people. Intermittent adherence to antidepressants may lessen their effectiveness and explain the relationship between wider antidepressant use and increased societal burden of depression.

## Introduction

The antidepressant era effectively began in 1953 with the observation that isoniazid elevated mood in people with tuberculosis (Lorenz et al., 1953). Since that time, many new antidepressants have been developed and marketed, and the rate of antidepressant prescription has risen substantially, year-on-year (Lalji et al., 2021).

Greater use of effective medications might have been anticipated to reduce the burden of depression on society such that workdays lost to depression might have fallen, for example. However, the opposite trend has been observed – the economic burden of depression has in fact increased despite wider use of antidepressants (Greenberg et al., 2021; Tanner et al., 2020). This growing economic burden might be rightly attributed to the ever-increasing rate of depression diagnosis in most Western Countries. Nonetheless, the availability of effective treatments for depression should mean that depression can be successfully treated and its economic burden minimised.

One explanation for the growth in the economic burden associated with depression is that antidepressants themselves are insufficiently effective. Concerns have been raised regarding the overall efficacy of antidepressants (Kirsch et al., 2008) and whether response to antidepressant treatment varies according to depression subtype or severity (Uher et al., 2011). It has also been suggested that antidepressants may actually worsen the conditions they treat (Fava, 2003).

However, there is good evidence that antidepressants are effective in the medium to long term (Lewis et al., 2021) and that taking antidepressants for a sufficient duration will prevent depression from returning (Kato et al., 2021; Liu et al., 2021). An alternative explanation is that how antidepressants are prescribed or taken in the real world renders them less effective than in the more controlled environment of clinical trials. Taking antidepressants only for short periods (Hunot et al., 2007) seems to predispose to relapse (Hirschfeld, 2001). Abruptly stopping antidepressants at any time worsens the risk of relapse (Baldessarini et al., 2010), although withdrawal symptoms may inflate the measured risk of relapse (Horowitz and Taylor, 2022).

Outside the well-ordered environment of a clinical trial, patients in the real world may stop and re-start antidepressant treatment; that is, they might be only partially adherent. It is conceivable that intermittent treatment resulting from partial adherence might lead to loss of efficacy and offer at least one explanation for the apparent underperformance of antidepressants. We sought to discover and summarise studies examining outcomes following discontinuation and restarting the same antidepressant.

## Method

In this systematic review, we searched PubMed and EMBASE databases for articles published from the beginning of the database records to the date the search was completed, 10 June 2024. Our search terms included a range of keywords that aimed to identify studies where antidepressants were discontinued and subsequently reinstated. We limited our search results to human studies.

The search terms used for PubMed were as follows: (‘Drug Tolerance’ [Mesh] OR tachyphyla* [tiab] OR non-respon* [tiab] OR nonrespon* [tiab] OR loss of response* [tiab] OR toleran* [tiab] OR re-initiation* [tiab] OR restart* [tiab] OR reproducib* [tiab] OR reinstate* [tiab]) AND (‘Depressive disorder’ [Mesh] OR depressive disorder* [tiab]) AND (‘Antidepressive Agents’ [Pharmacological Action] OR ‘Antidepressive Agents’ [Mesh] OR ‘Serotonin Uptake Inhibitors’ [Mesh] OR antidepressiv* [tiab] OR antidepressant* [tiab] OR SSRI* [tiab] OR SNRI* [tiab] OR TCA* [tiab] OR serotonin reuptake inhibitor* [tiab]) NOT (‘Animals’ [Mesh] NOT (‘Animals’ [Mesh] AND ‘Humans’ [Mesh])). The search terms used for EMBASE were: (drug tolerance.mp. or exp drug tolerance or tachyphylaxis.mp. or nonrespon*.mp. or non-respon*.mp. or re-initiation*.mp. or restart*.mp. or reproducib*.mp. or reinstate*.mp.) AND (depressive disorder.mp. or exp depression/or major depression*.mp.) AND (anti-depressive agent.mp. or exp antidepressant agent/or serotonin reuptake inhibitors.mp. or exp serotonin uptake inhibitor/or antidepressive*.mp. or antidepressant*.mp. or SSRI*.mp. or SNRI*.mp. or TCA*.mp.) AND limit to humans.

Records were screened for duplicates electronically initially, after which a manual check for duplicates was conducted. We included articles that reported data on clinical outcomes after the reinstatement of antidepressants after discontinuation. Studies that reported antidepressant reinstatement but did not report clinical outcomes were not included. We did not restrict studies included in our review based on psychiatric diagnosis. Review articles, correspondence and editorials on the topic of antidepressant discontinuation were screened for further references for inclusion in the review.

## Results

**Figure 1.**
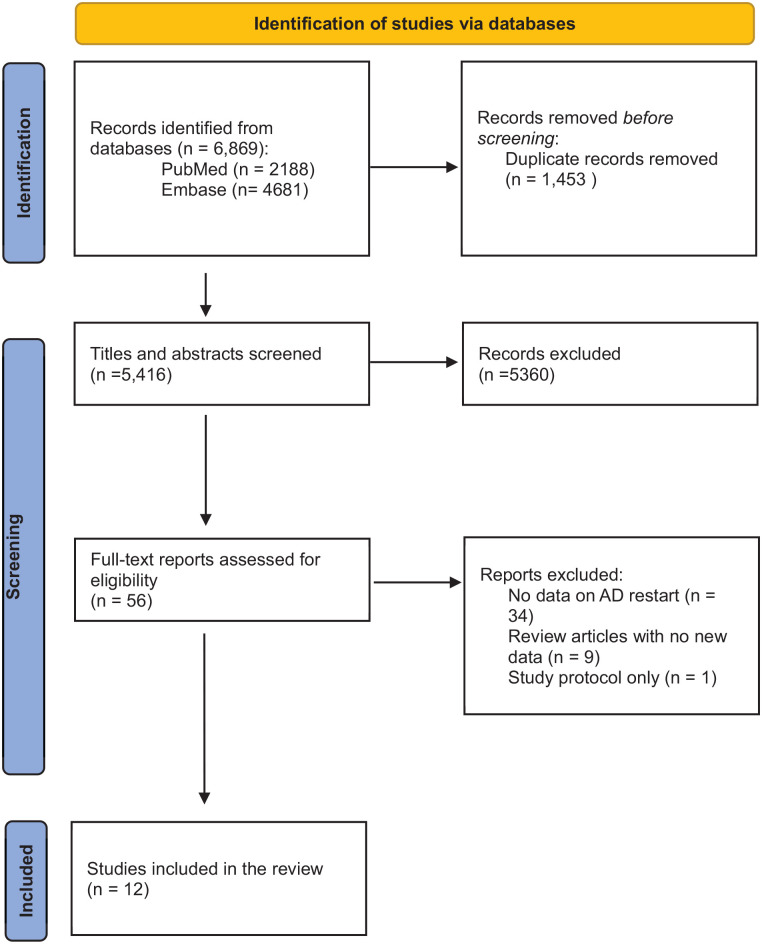
Systematic literature search PRISMA flow diagram.

The results of the search strategy are outlined in Figure 1. We identified a total of 6869 articles from our literature search. After accounting for duplicates and screening titles and abstracts, we screened 56 full-text articles for inclusion in this review. Following the full-text screen, 12 articles met the inclusion criteria for this review. Articles written in a non-English language were included for screening, but none was eligible for inclusion (Table 1).

**Table 1.** Characteristics of included studies.

Study	*n*	Antidepressant	Methods used to assess depression severity	Duration of taper	Duration of AD-free period	Outcome (detail)	Outcome % non-responsive after restarting
Fava et al. (2006)	58	Duloxetine	HAMD, CGI	1 week	Varied up to 26 weeks	Of 58 who reinstated treatment with duloxetine 60 mg, 74% (*n* = 43) ‘responded’ and 57% (*n* = 32) achieved ‘remission’	26%
Fava et al. (2002)	55	Fluoxetine	HAMD, CGI	None	Varied up to 25 weeks	Of 55 who reinstated treatment with fluoxetine 20 mg, 6 never responded, 14 responded but did not maintain response over 6 months, and 33 responded and maintained response over 6 months	11%
Flint and Rifat (1999)	12	Nortriptyline, phenelzine	HAMD, HADS, MMS, LEDS, Burvill scale	Up to 8 weeks	Varied, up to 2 years	Of 11 patients who restarted treatment with antidepressants, 10 responded, one of whom had to restart adjunctive lithium to achieve response	8%
Yoshimura et al. (2022)	23	Escitalopram	HAMD	6 months or more	Varied, mean was 12.8 (SD 7.8) months	Of 23 patients who reinstated escitalopram treatment, 10 patients responded, 6 of whom achieved remission at week 8. Thirteen patients did not respond to escitalopram reinstatement	57%
Friedman et al. (1995)	12	Desipramine	HAMD, SCID, GAS	None	Varied, up to 114 weeks	Of 12 patients who restarted treatment with desipramine, 11 achieved full remission by 8 weeks	8%
Maina et al. (2001)	81	Clomipramine (*n* = 18), fluoxetine (*n* = 22), fluvoxamine (*n* = 21), paroxetine (*n* = 20)	SCID, Y-BOCS, HAMD	1 week	Up to 6 months	Of 81 patients that restarted treatment, 68 patients responded to the reinstatement of antidepressant at 6 months	16%
Versiani et al. (1997)	51	Moclobemide	SCID, CGI-S, CGI-C, LAS, HAMA, HAMD	Unclear	2–4 months	Of 51 patients who restarted moclobemide, 49 responded to treatment	4%
Reynolds et al. (1994)	32	Nortriptyline	HAMD, GAS	None	Mean of 2 weeks	Of 32 patients who were assigned retreatment with nortriptyline, 27 responded. Two did not complete treatment, and 3 completed treatment and did not respond	19% (ITT analysis), 10% (completed treatment)
Remillard et al. (1994)	23	Tricyclics (*n* = 18), trazodone (*n* = 2), amoxapine (*n* = 2), nomifensine (*n* = 1)	Response measured with screening notes for phrases, for example, ‘improved’, no formal scale	No taper	Not mentioned	Of the 23 patients who received an adequate trial of the previously effective antidepressant, 16 responded. For all patients who received the same antidepressant as previously (including inadequate trials), of 35 patients, 20 responded	30% (for adequate trials)
Fava et al. (1998)	47	Unclear, in the previous study protocol, but not reported	Unclear	Equivalent to amitriptyline 25 mg every other week	Not reported	Of 47 patients who received the same antidepressant following relapse, 45 responded	4%
Rosenbaum et al. (1998)	192	Fluoxetine, paroxetine, sertraline	MADRS, HDRS28, SQ, DESS	No taper	5–8 days (treatment break)	No data on categories of ‘response’. Differences in HAMD and MADRS scores are reported in the article. No significant differences in endpoint HAMD and MADRS scores in those that interrupted their treatment for 5–8 days with placebo versus those that maintained on treatment	No ‘response’ definition. Mean HAMD and MADRS scores did not change
Roca et al. (2013)	40	SSRIs	EPDS, STAI	None	Varied, up to 36 weeks	EPDS scores calculated at first visit (before 20 weeks) and at prenatal visit (34–36 weeks). In 62 patients who maintained SSRI: First visit: 12.1 (SD 6.8), prenatal visit: 8.1 (SD 6.2). In 40 patients who stopped and reintroduced: FV: 18.8 (SD 6.6), PV: 13.5 (SD 7.2). In 30 patients who stopped and did not reintroduce: FV: 12.7 (SD 7.0), PV: 9.6 (SD 4.3)	No ‘response’ definition, but patients reintroducing SSRIs had a higher EPDS than those who maintained treatment at the prenatal visit

Of the 12 included studies, 10 expressed numbers of patients ‘responsive’ after reinstating antidepressant treatment. Of these 10 studies, 5 included no tapering of antidepressant dose when discontinuing the antidepressant. Nine included studies examined the treatment of depression (as it was defined at the time of each study), one OCD, one dysthymia and one social phobia with GAD.

### Narrative description of included studies

This was a multisite double-blind placebo-controlled RCT in patients with major depressive disorder. Patients who responded to 60 mg duloxetine after 12 weeks of treatment were randomised to placebo or continuation with duloxetine. In the placebo group, the dose of duloxetine was reduced to 30 mg for 1 week and then stopped. Rescue treatment at the previous dose of duloxetine was used if a relapse occurred. Overall, 26% of patients relapsed and then did not respond to reinstated duloxetine (Fava et al., 2006).

This was a multisite, double-blind, placebo-controlled RCT of responders to fluoxetine 20 mg a day. Those randomised to placebo had fluoxetine abruptly stopped, and restarted if a relapse occurred. Overall, 11% of participants did not respond at all to fluoxetine on reinstatement after relapse, and 25% of those who previously maintained a response in the initial treatment episode did not maintain a response after restarting treatment (Fava, 2003).

This open-label study investigated the recurrence of depression and the effect of restarting treatment in older patients with a first episode of major depression. After a tapering period of 8 weeks, patients were followed up for 2 years, and their antidepressant was restarted if depression recurred. Most relapses occurred within 1 year. One of 11 relapsing patients did not respond on re-exposure (Flint and Rifat, 1999).

Investigators described an open-label trial where patients in remission from a first episode of major depression discontinued escitalopram over a period of 6 months or more. If depression recurred, escitalopram was restarted. Overall, 43% of patients responded to escitalopram on re-exposure (i.e. 57% did not respond). The authors noted that the mean reduction in HAMD was greater with the initial treatment than with the restarted treatment (Yoshimura et al., 2022).

This was an open-label antidepressant restarting phase of a double-blind RCT in patients who met the criteria for DSM-III dysthymia before initial treatment with desipramine. Responders to desipramine were randomised in a double-blind manner to receive either placebo or to continue desipramine. If relapse occurred, then open-label treatment with desipramine was restarted. One of 12 patients who relapsed was unresponsive when desipramine was restarted (Friedman et al., 1995).

This open-label trial reported initial antidepressant treatment and restarting of antidepressant treatment in patients with obsessive-compulsive disorder. Patients were restarted on their original treatment at the dose that was previously effective for them if relapse occurred during a 6-month follow-up. The authors reported a statistically significant difference in response to antidepressant treatment when comparing the initial treatment episode with their corresponding reinstitution episode. Thirteen of 81 patients (16%) did not respond at all on restarting (Maina et al., 2001).

This open, prospective naturalistic study explored moclobemide treatment in patients with social phobia and concurrent generalised anxiety disorder or mood disorder. After abrupt discontinuation, treatment was restarted if social phobia recurred. Within 9 months, 96% of those in whom treatment was restarted responded. Response was defined as a CGI-C scale score of <3 (moderate or substantial improvement; Versiani et al., 1997).

In this study, older patients with a first episode of depression were treated with nortriptyline and interpersonal psychotherapy. Patients were then randomised to psychotherapy plus placebo or placebo alone. All those randomised to placebo following effective treatment were monitored for relapse, and treatment was restarted if necessary. The mean time to restarting treatment was 2 weeks. Of those having an initial adequate trial of treatment, 3 of 32 (10%) did not respond on restarting (Reynolds et al., 1994).

The study by Remillard and others was an observational study exploring records of patients who experienced two episodes of depression and received antidepressant treatment as inpatients for these episodes. The authors’ criteria for response were the use of phrases such as ‘improved’ or ‘recovered’ in the patient’s notes and discharge from the hospital. In total, 30% of people did not respond to reinstatement after relapse (Remillard et al., 1994).

Investigators of a randomised, open-label study on major depressive disorder and cognitive behavioural therapy (CBT) reported outcomes of discontinuation of antidepressants in patients previously effectively treated with antidepressants (Fava et al., 1998). Participants were randomised to either CBT or clinical management. If participants experienced a recurrence of major depression, their previously effective antidepressant was restarted. In total, 4% of participants failed to respond on restarting after having relapsed.

The study by Rosenbaum and co-workers investigated the emergence of discontinuation events and depressive symptoms in patients effectively treated with antidepressants for unipolar depression and in whom treatment was briefly discontinued. All participants had their treatment with antidepressants abruptly interrupted for up to 8 days before restarting treatment. Patients were visited weekly before, during and after treatment interruption. HAMD scores showed no significant difference before and after treatment interruption, although HAMD scores were very much greater during treatment interruption in patients prescribed sertraline and paroxetine (but not fluoxetine). There seemed to be no loss of therapeutic effect after removal of treatment for 1 week. The brevity and abruptness of the interruption suggest that changes in HAMD scores were caused by withdrawal symptoms rather than relapse per se (Rosenbaum et al., 1998).

Roca and colleagues prospectively examined the treatment of women with unplanned pregnancy referred to a perinatal psychiatry service. Participants were patients with depressive or anxiety disorders treated effectively with antidepressants at the time of conception. Treatment, if discontinued, was discontinued when pregnancy was discovered. Patients who discontinued and subsequently restarted treatment with antidepressants were followed up. Edinburgh Perinatal Depression Scale (EPDS) scores were reported for participants at their first visit and their prenatal visit. The time off treatment varied from a few weeks to several months. EPDS scores were greater in those who interrupted antidepressant treatment during pregnancy than in those who continued, indicating some loss of effectiveness (Roca et al., 2013).

## Discussion

We found data on 594 participants who stopped and then restarted an individual antidepressant with a gap in treatment of between 5 days and greater than 2 years. Of 10 studies reporting failure rates on re-exposure to an antidepressant (*n* = 362), non-response occurred in 4%–57% of participants. In studies not reporting response rates, there was evidence of reduced magnitude of response on restarting. Given these observations, there is some certainty that breaks in treatment can lead to loss of antidepressant response. However, there is much less certainty about the factors that influence future non-responsiveness and the degree to which response is lost.

Interpretation of reported outcomes is made difficult by several considerations. Most of these studies included either no tapering period or a very rapid tapering of antidepressant treatment, whereas some had prolonged tapering periods. Risk of non-response did not seem to be associated with the tapering method. In fact, the only study in which participants showed no loss of responsiveness was one in which antidepressants were deliberately withdrawn abruptly (Rosenbaum et al., 1998). However, in this study, participants did not relapse, as such, but merely showed inflated depression rating scale scores because of the presence of withdrawal symptoms. Thus, the effect of reinstatement was to cause withdrawal symptoms to abate rather than to re-exert an antidepressant effect. For many studies, it is much less clear whether antidepressants were reinstated because of relapse of the disorder for which they initially received treatment or because antidepressant withdrawal symptoms mimicked relapse. The study with the longest withdrawal period (Yoshimura et al., 2022) had the highest non-response rate (57%), perhaps reflecting the absence of reinstatement effects on withdrawal symptoms. It is also possible that reinstatement initially mitigates withdrawal symptoms but does not provide longer-term antidepressant or other activity – this seems to have been demonstrated by Fava (2003) who observed a reduced maintenance of response in patients who had previously discontinued fluoxetine abruptly.

These findings raise the possibility that the intermittent taking of antidepressants (e.g. in partial adherence) leads both to loss of efficacy and some loss of future responsiveness. People prescribed antidepressants are more likely to stop treatment than to continue for 6 months (Sawada et al., 2009), and this is often done without the awareness of the prescriber (Woodward et al., 2016). Patient-determined antidepressant cessation is likely to be abrupt and to occur before the end of the recommended treatment period. Both of these factors predict depressive relapse. As our study shows, any hiatus in treatment seems to confer a risk of loss of response. Thus, these three factors combine to worsen outcomes in depression and may go some way to explain the increasing burden of depression in society despite the ready availability and widespread prescribing of antidepressants. There is a need for formulations to be developed that assure delivery of antidepressant treatment, perhaps in the same way that long-acting injectable formulations are used in schizophrenia and HIV treatment.

The main strength of this study is the systematic and wide-ranging literature review that was conducted, meaning that we are likely to have uncovered all relevant publications. The main weakness is that we found data on only just over 500 patients whose treatment was prescribed for various conditions, stopped for different periods and withdrawn in different ways. There was little uniformity in response evaluation. A secondary weakness is that we did not examine what mechanism might lie behind the loss of response to an individual antidepressant after an interruption in treatment.

## Conclusion

Interrupting antidepressant treatment confers an increased risk of non-response. Abrupt discontinuation and brief treatment periods increase the risk of depressive relapse. These three factors may combine to explain the growing burden of depression on society in spite of the greater use of antidepressants.
